# Investigating the relationship between depression and breast cancer: observational and genetic analyses

**DOI:** 10.1186/s12916-023-02876-w

**Published:** 2023-05-04

**Authors:** Xueyao Wu, Wenqiang Zhang, Xunying Zhao, Li Zhang, Minghan Xu, Yu Hao, Jinyu Xiao, Ben Zhang, Jiayuan Li, Peter Kraft, Jordan W. Smoller, Xia Jiang

**Affiliations:** 1 Department of Epidemiology and Health Statistics, West China School of Public Health and West China Fourth Hospital, Sichuan University, No. 16 Ren Min Nan Lu, Chengdu, Sichuan 610041 People’s Republic of China; 2 Department of Biostatistics, Harvard T.H. Chan School of Public Health, Boston, MA USA; 3 Department of Epidemiology, Harvard T.H. Chan School of Public Health, Boston, MA USA; 4 Psychiatric and Neurodevelopmental Genetics Unit, Center for Genomic Medicine, and Department of Psychiatry, Massachusetts General Hospital, MA Boston, USA; 5 Stanley Center for Psychiatric Research, Broad Institute of MIT and Harvard, MA Cambridge, USA; 6 Department of Clinical Neuroscience, Center for Molecular Medicine, Karolinska Institutet, Stockholm Solna, Sweden; 7 Department of Nutrition and Food Hygiene, West China School of Public Health and West China Fourth Hospital, Sichuan University, Chengdu, China

**Keywords:** Depression, Breast cancer, Longitudinal association, Genetic correlation, Pleiotropic loci, Causal inference

## Abstract

**Background:**

Both depression and breast cancer (BC) contribute to a substantial global burden of morbidity and mortality among women, and previous studies have observed a potential depression-BC link. We aimed to comprehensively characterize the phenotypic and genetic relationships between depression and BC.

**Methods:**

We first evaluated phenotypic association using longitudinal follow-up data from the UK Biobank (*N* = 250,294). We then investigated genetic relationships leveraging summary statistics from the hitherto largest genome-wide association study of European individuals conducted for depression (*N* = 500,199), BC (*N* = 247,173), and its subtypes based on the status of estrogen receptor (ER + : *N* = 175,475; ER − : *N* = 127,442).

**Results:**

Observational analysis suggested an increased hazard of BC in depression patients (HR = 1.10, 95%CIs = 0.95–1.26). A positive genetic correlation between depression and overall BC was observed (${r}_{g}$ = 0.08, *P* = 3.00 × 10^–4^), consistent across ER + (${r}_{g}$ = 0.06, *P* = 6.30 × 10^–3^) and ER − subtypes (${r}_{g}$ = 0.08, *P* = 7.20 × 10^–3^). Several specific genomic regions showed evidence of local genetic correlation, including one locus at 9q31.2, and four loci at, or close, to 6p22.1. Cross-trait meta-analysis identified 17 pleiotropic loci shared between depression and BC. TWAS analysis revealed five shared genes. Bi-directional Mendelian randomization suggested risk of depression was causally associated with risk of overall BC (OR = 1.12, 95%Cis = 1.04–1.19), but risk of BC was not causally associated with risk of depression.

**Conclusions:**

Our work demonstrates a shared genetic basis, pleiotropic loci, and a putative causal relationship between depression and BC, highlighting a biological link underlying the observed phenotypic relationship; these findings may provide important implications for future studies aimed reducing BC risk.

**Supplementary Information:**

The online version contains supplementary material available at 10.1186/s12916-023-02876-w.

## Background

Individuals with mental health disorders are often at an increased risk for an array of subsequent medical conditions including cancer [1]. One example of this phenotypic association is depression and breast cancer (BC), both of which disproportionately or primarily affect women and contribute to a substantial global burden of morbidity and mortality [2, 3]. Hypothesized underlying biological mechanisms include enhanced inflammation and oxidative stress, inhibited immune surveillance, and dysfunctional activation of the autonomic nervous system and the hypothalamic–pituitary–adrenal axis [1]. Population-based evidence regarding the depression-BC relationship, however, remains inconsistent. While one meta-analysis aggregating data from 11 cohort studies involving 182,241 individuals identified an increased (though non-significant) risk of BC among individuals with depression (pooled relative risk (RR) = 1.13, 95% confidence intervals (95%CIs) = 0.94–1.36) [4], another meta-analysis restricted to cohort studies with follow-up periods longer than ten years reported a significant association (pooled RR = 2.50, 95%CIs = 1.06–5.91) [5]. Nevertheless, phenotypic correlations derived from observational studies can be subject to bias, confounding, and reverse causality [6].

One way to disentangle these conflicting findings is to investigate the potential genetic underpinnings of comorbid disorders. Twin studies have established that both depression and BC are under genetic influence, with heritability estimates of 37% and 31%, respectively [7, 8]. More recently, large-scale genome-wide analysis of common variants have found significant genetic correlations between depression and multiple female reproductive phenotypes [9–11] including age at menarche and age at natural menopause (well-established risk factors for BC [12]). Multiple loci have further been identified (assessed in GWAS catalog [13] on Jan 15, 2023) as influencing both traits (i.e., *TENM2*, *BTN2A1*, *ESR1*, *ASTN2*, *SLC6A15*). These results suggest that depression and BC may be linked by shared biology, though the extent and nature of such links remains unclear.

**Fig. 1:**
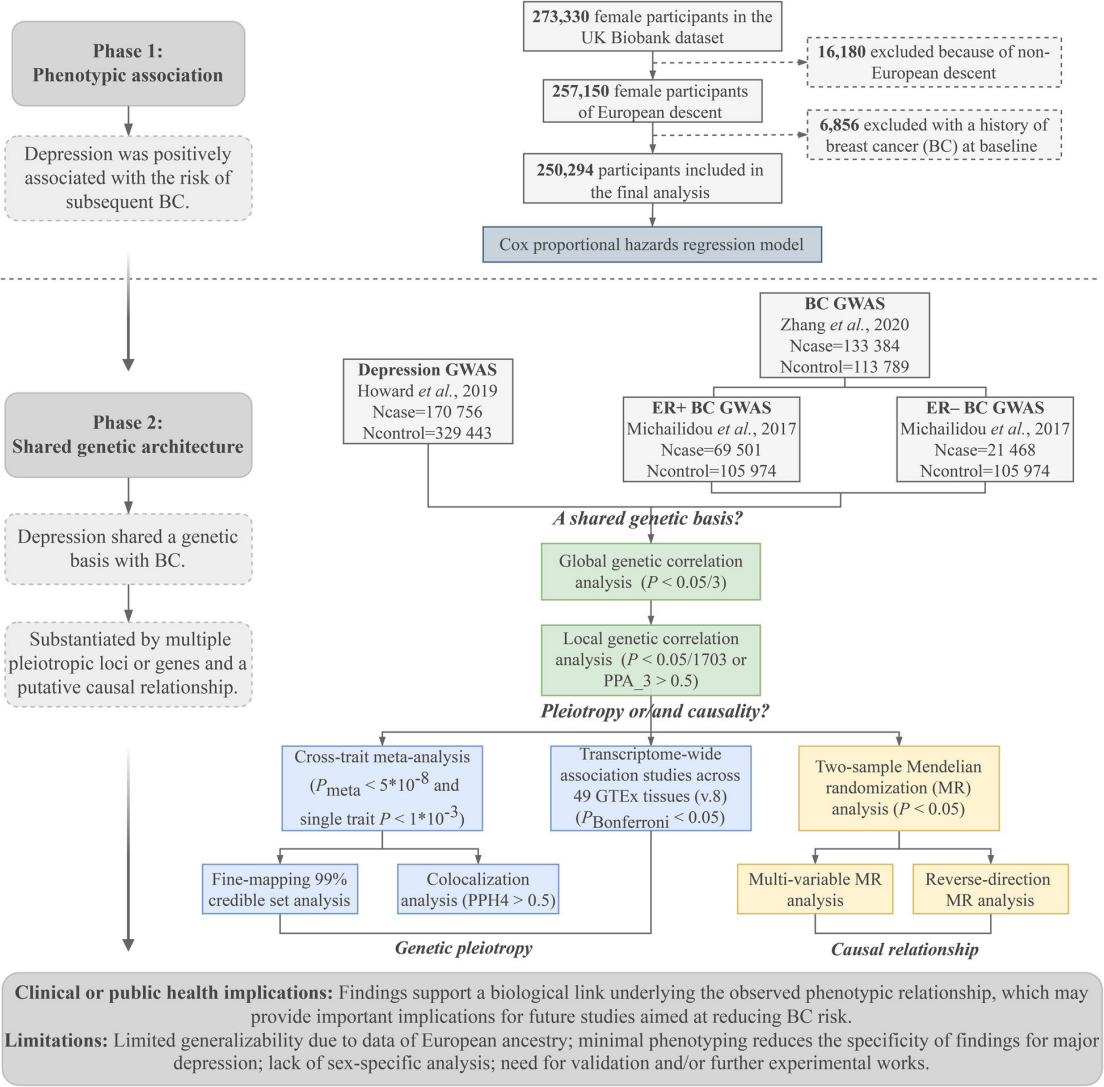
Flowchart of the overall study design. We first used longitudinal follow-up data from the UK Biobank to evaluate the phenotypic association between depression and breast cancer. We then leveraged summary statistics from the hitherto largest genome-wide association study conducted for each trait to characterize the shared genetic architecture through a genome-wide cross-trait analysis. BC: breast cancer; ER: estrogen receptor; GWAS: genome-wide association study; GTEx: Genotype-Tissue Expression project; MR: Mendelian randomization

Recent advances in statistical genetics have yielded a range of methods to enable comprehensive genome-wide cross-trait analyses to characterize the shared and distinct genetic influences across traits, driving forward epidemiologic associations with novel insights into the underlying biological mechanisms [6]. Here we apply these methods to dissect the genetic and phenotypic relationships between depression and BC. Specifically, leveraging the hitherto largest observational and genetic data, we quantified phenotypic association, global and local genetic correlations, pleiotropic loci, and potential causal relationships. Given the role estrogen receptor (ER) plays in the pathogenesis of depression [14], we further investigated the shared genetic architecture with depression across BC subtypes characterized by distinct ER status. Figure 1 illustrates the overall design of the study, with Additional file 1: Figure S1 providing an accompanying graphical abstract.

## Methods

We first used data from UK Biobank (UKBB) to evaluate the phenotypic association. We then leveraged summary statistics from genome-wide association study (GWAS) conducted for each trait to characterize the shared genetic architecture through a genome-wide cross-trait analysis. The Strengthening the Reporting of Observational Studies in Epidemiology (STROBE) checklists for both observational and genetic studies are detailed in Additional file 2.

### Data sources

#### UK Biobank data

UKBB is a large-scale prospective cohort study with more than 500,000 UK participants (55% women) aged 40–69 years when recruited in 2006–2010 [15]. At recruitment, all participants gave informed consent for participation and follow-up. Overall, 503,317 participants consented to join the study cohort and visited an assessment center, among which we only considered women of European descent (N = 257,150). We defined a diagnosis of depression as the ICD-10 (international classification of diseases, 10th revision) codes F32, F33, F34, F38, and F39, and a diagnosis of BC as the ICD-10 code C50 and the ICD-9 code 174, using linked records of hospital admission in UKBB (Data-Field: 41,202 and Data-Field: 41,204). After excluding 6,856 participants with a history of BC at baseline, 250,294 participants were finally included.

#### GWAS datasets

GWAS summary data for depression was obtained from a meta-analysis of 807,553 individuals (246,363 cases and 561,190 controls, all of European ancestry) combining the three largest existing genetic studies of depression (UKBB, 23andMe and the Psychiatric Genomics Consortium (PGC)) [9]. The phenotypes ranged from self-reported help-seeking for problems with “nerves, anxiety, tension or depression” (termed “broad depression”) (51.8%), self-reported clinical diagnosis of major depression (MD) (30.7%), and clinically ascertained diagnosis of MD (17.5%). Independent trait-associated genome-wide significant single nucleotide polymorphisms (SNPs) were identified at a *P*-threshold of 5 × 10^–8^, after clumping SNPs in linkage disequilibrium (LD) (${r}^{2}$ > 0.10 across a 3.0 Mb window) with the top-associated SNPs.

We extracted the effect size and relevant information of the 102 GWAS-identified depression-associated significant SNPs and used those SNPs as instrumental variables (IVs) in Mendelian randomization (MR) analyses [16] (Additional file 3: Table S1). We also retrieved the full set of summary statistics for depression, in which data from 23andMe were excluded (due to limited data availability).

The largest available GWAS summary data for overall BC was obtained from a meta-analysis of 247,173 individuals (133,384 clinically ascertained BC cases and 113,789 controls, all of European ancestry), combining data from 82 participating studies of the Breast Cancer Association Consortium (BCAC) and 11 other BC genetic studies [17]. Top-associated SNPs in the combined meta-analysis reaching a *P*-threshold of 5 × 10^–8^ were reported. This GWAS identified 32 novel susceptibility loci in addition to 153 previously detected loci (for a total of 185 genome-wide significant loci). For subtype-specific BC, the hitherto largest GWAS summary data was obtained from a meta-analysis of 122,977 BC cases (69,501 ER + BC and 21,468 ER − BC) and 105,974 controls (all of European ancestry) combining data from BCAC and DRIVE (Discovery, Biology and Risk of Inherited Variants in Breast Cancer Consortium) [18]. No overlapping participating studies were shared between the BCs and depression GWASs.

We extracted the effect size and relevant information of the 185 GWAS-identified BC-associated significant SNPs for reverse-direction MR analysis. As trait-associated SNPs were not reported for subtype-specific BC, we determined IVs as the lead SNPs reaching genome-wide significance (*P* < 5 × 10^–8^) after removing SNPs in LD (${r}^{2}$ > 0.001 across a 10.0 Mb window). We also downloaded full summary statistics for all BCs (overall and subtypes).

Details on the characteristics of each included data set are presented in Additional file 3: Table S2.

### Statistical analysis

#### Observational analysis

We constructed a Cox proportional hazards regression model with exposure to depression modeled as a time-dependent variable. We used three sets of adjustments to minimize the role of confounding. Estimates in model 1 were adjusted only for age, assessment center, and the top 40 genetic principal components. Estimates in model 2 were adjusted additionally for income, Townsend deprivation index, body mass index (BMI), smoking, drinking, physical activity, sleep duration, and education. Estimates in model 3 were adjusted, on top of model 2, for family history of BC, reproductive factors (parity, age at menarche, menopausal status, use of oral contraceptives, and hormone replacement therapy), diagnosis of other mental health disorders, and treatment/medicine for antidepressants or antipsychotics. All analyses were conducted using SAS version 9.4 (SAS Institute, Cary, NC).

#### Global genetic correlation analysis

To evaluate an overall shared genetic basis between depression and BC, we estimated their global genetic correlation using cross-trait LD Score Regression (LDSC) [19]. LDSC requires only GWAS summary statistics as input and quantifies the average sharing of genetic effect between pairs of traits. The genetic correlation (${r}_{g}$) ranges from –1 to 1, with –1 indicating a perfect negative correlation and 1 indicating a perfect positive correlation. A Bonferroni corrected *P*-value of 0.017 was used as significant threshold (*P* < 0.05/3, number of overall BC and subtypes).

#### Local genetic correlation analysis

To identify genomic regions that contribute disproportionately to the global genetic correlation between depression and BC, we further estimated local genetic correlation in 1,703 pre-defined LD-independent regions using both Heritability Estimator from Summary Statistics (ρ-HESS) and Pairwise-GWAS (GWAS-PW) [20, 21]. While ρ-HESS provides a precise quantification of each genomic region, GWAS-PW uses a Bayesian framework to calculate the posterior probabilities of association (PPA) of a genomic region under four models [21]. Significant local signal was determined if the *P*-value from ρ-HESS survived multiple corrections (*P*_ρ-HESS_ < 0.05/1703), or if PPA of model 3 (PPA _3, the probability that a genomic region associated with both traits) from GWAS-PW was larger than 0.5.

#### Cross-trait meta-analysis

Significant genetic correlation either suggest genetic variants having independent effect on both traits (pleiotropy) or genetic variants influencing one trait via its effect on the other (causality). We next performed a cross-trait meta-analysis using Cross-Phenotype Association (CPASSOC) to identify pleiotropic loci [22]. Based on the fixed-effect model, CPASSOC provides two estimates, S_Hom_ and S_Het_, to combine summary statistics across traits while controlling population structure and cryptic relatedness. S_Hom_ is the most powerful when genetic effect sizes are homogenous, which is unlikely to be true when meta-analyzing multiple traits. As an extension of S_Hom_, S_Het_ maintains statistical power even under the presence of heterogeneity by assigning more weights to the larger trait-specific effect sizes, and was thus adopted for the analysis herein.

We applied PLINK’s “clumping” function to obtain independent loci (parameters: –clump-p1 5e-8 –clump-p2 1e-5 –clump-r2 0.2 –clump-kb 500) [23]. Within each locus, the variant with the lowest *P*-value was kept as index SNP. Significant pleiotropic SNPs were defined as index variants satisfying *P*_CPASSOC_ < 5 × 10^–8^ and *P*_single-trait_ < 1 × 10^–3^ (for both traits). Novel pleiotropic SNPs were defined as significant pleiotropic SNPs that did not reach genome-wide significance in single-trait GWASs (5 × 10^–8^ < *P*_single-trait_ < 1 × 10^–3^), were independent (${r}^{2}$< 0.20) of previously reported genome-wide significant SNPs (of depression and BC), and none of their neighboring SNPs (± 500 kb) reached *P* < 5 × 10^–8^ in single-trait GWASs.

We used Ensembl Variant Effect Predictor (VEP) [24] and 3DSNP [25] for detailed functional annotation of the identified pleiotropic SNPs. While VEP selects candidate genes based on simple physical proximity [24], 3DSNP annotates the regulatory function of SNPs by exploring their 3D interactions with genes mediated by chromatin loops [25].

#### Fine mapping credible set analysis

Because an index SNP is not likely to be the causal SNP, we further identified a credible set of variants that were 99% likely to contain causal variants at each of the pleiotropic loci through FM-summary (https://github.com/hailianghuang/FM-summary) [26]. Briefly, FM-summary is a Bayesian fine-mapping algorithm that maps only the primary signal and uses a flat prior with steepest descent approximation, assuming at least one causal variant exists within a given region.

#### Colocalization analysis

To investigate whether the same variants are responsible for two GWAS signals as opposed to distinct genetic variants in proximity, we next performed a colocalization analysis using Coloc [27]. This method provides posterior probabilities for five mutually exclusive hypotheses regarding the sharing of causal variants in a genomic region. We extracted summary statistics for variants within 500 kb of each shared index SNP, and calculated the posterior probability for H4 (PPH4, the probability that both traits associated through sharing a single causal variant). A locus was considered colocalized if PPH4 was greater than 0.5.

#### Transcriptome-wide association studies

Many genetic variants influence complex traits by modulating gene expression; thus, investigating overlapped genes may help clarify causal mechanisms. To identify associations between depression and BC with regard to gene expression in specific tissues, we conducted a transcriptome-wide association study (TWAS) using FUSION [28] by integrating GWAS summary data with expression weights across 49 tissues from GTEx (Genotype-Tissue Expression, version 8). We first performed 49 TWASs for each trait, one tissue-trait pair at a time, to identify an independent set of gene-tissue pairs. We then intersected single-trait TWAS results to examine if there were shared gene-tissue pairs across traits. The Bonferroni correction was used within each tissue to account for multiple comparisons.

#### Mendelian randomization analysis

We finally performed a comprehensive two-sample MR analysis to detect potential causal relationships. We used the random-effect inverse-variance weighted (IVW) approach as the primary approach [29]. This method pools the Wald ratio estimate of each SNP and obtains the average casual effect estimate between two traits. To reduce biased estimate due to pleiotropic effects of genetic instruments, we adopted two complementary methods: MR-Egger regression (detects and corrects for bias due to directional pleiotropy) [30], and weighted median approach (provides a consistent causal estimation with even ≥ 50% invalid IVs) [31]. As these two approaches were less powerful than IVW in detecting true causal effects, we defined a significant causal estimate as significant in IVW (*P*-value < 0.05/3, number of overall BC and subtypes) and showing directional consistency in MR-Egger regression and weighted median approach.

Additional sensitivity analyses were conducted to validate MR model assumptions (i.e., relevance, independence, and exclusion restriction) [32], including: (i) exclusion of palindromic IVs with strand ambiguity; (ii) exclusion of pleiotropic IVs associated with potential confounding traits (confirmed by GWAS Catalog [13]); (iii) leave-one-out analysis where one SNP was removed at a time and IVW was conducted based on the remaining SNPs; and (iv) MR-Pleiotropy Residual Sum and Outlier (MR-PRESSO) to evaluate the presence of horizontal pleiotropy and to re-calculate causal effects after removing the detected outliers [33]. A multi-variable MR (MVMR) approach [34] was further employed to account for the effect from major confounders, including BMI [35], smoking initiation [36], alcohol consumption [36], physical activity [37], sleep duration [38], and educational attainment [39], where confounders were incorporated together with depression, one at a time as well as simultaneously. To evaluate whether genetically predicted BC exerts a causal effect on depression, a reverse-direction MR was performed where BC-associated independent SNPs were used as IVs.

MR analyses were conducted using packages “TwoSampleMR” (version 0.5.4), “MRPRESSO” (version 1.0), and “MendelianRandomization” (version 0.7.0) in software R (version 4.1.0).

## Results

### Phenotypic association

Baseline characteristics of UKBB participants included in the observational analysis are presented in Additional file 3: Table S3. In total, participants were followed for 3,014,168 person-years (11.41 ± 2.95 years), during which 529 depression patients and 9,516 depression-free individuals developed BC (Table 1). After adjusting for age, assessment center, and the top 40 genetic principal components, we observed a positive association between depression and risk of BC (hazard ratio (HR) = 1.075, 95%CIs = 0.963–1.199). The effect was strengthened when additionally adjusted for income, Townsend deprivation index, and lifestyle-related factors (HR = 1.090, 95%CIs = 0.951–1.250). In the fully adjusted model, the effect stabilized to 1.096 (95%CIs = 0.951–1.264).

**Table 1:** Observational association between depression and breast cancer

Depression status during following up	Breast cancer cases/person years	Model 1	Model 2	Model 3
No	9,516/2,912,715	1.000 (ref)	1.000 (ref)	1.000 (ref)
Yes	529/101,453	1.075 (0.963–1.199)	1.090 (0.951–1.250)	1.096 (0.951–1.264)

Model 1: adjusted for age, assessment center, and the top 40 genetic principal components

Model 2: adjusted for age, assessment center, the top 40 genetic principal components, income, Townsend deprivation index, body mass index, smoking, drinking, physical activity (IPAQ), sleep duration, and education

Model 3: adjusted for age, assessment center, the top 40 genetic principal components, income, Townsend deprivation index, body mass index, smoking, drinking, physical activity (IPAQ), sleep duration, education, family history of breast cancer, parity, age at menarche, menopausal status, use of oral contraceptives, hormone replacement therapy, diagnosis of other mental health disorders (i.e., bipolar disorder, multiple personality disorder, or schizophrenia/psychosis), and treatment/medication for antidepressants or antipsychotics

### Global and local genetic correlation

A limited but significant positive genetic correlation was found for depression with overall BC (${r}_{g}$ = 0.08, *P* = 3.00 × 10^–4^), which was also observed in both ER + (${r}_{g}$ = 0.06, *P* = 6.30 × 10^–3^) and ER − subtypes (${r}_{g}$ = 0.08, *P* = 7.20 × 10^–3^), all withstanding Bonferroni correction (Table 2).

**Table 2:** Global genetic correlation between depression and breast cancer

**Trait 1**	**Trait 2**	${{\varvec{r}}}_{{\varvec{g}}}$	${{\varvec{r}}}_{{\varvec{g}}}$ **_se**	${{\varvec{r}}}_{{\varvec{g}}}$ ***_P***	**gcov**	**gcov_se**
Depression	Overall breast cancer	0.08	0.0213	3.00 × 10^–4^	0.0102	0.0028
	ER + breast cancer	0.06	0.0231	6.30 × 10^–3^	0.0093	0.0034
	ER– breast cancer	0.08	0.0311	7.20 × 10^–3^	0.0107	0.0039

*r*_*g*_ Genetic correlation, *se* Standard error, *gcov* Genetic covariance

**Fig. 2:**
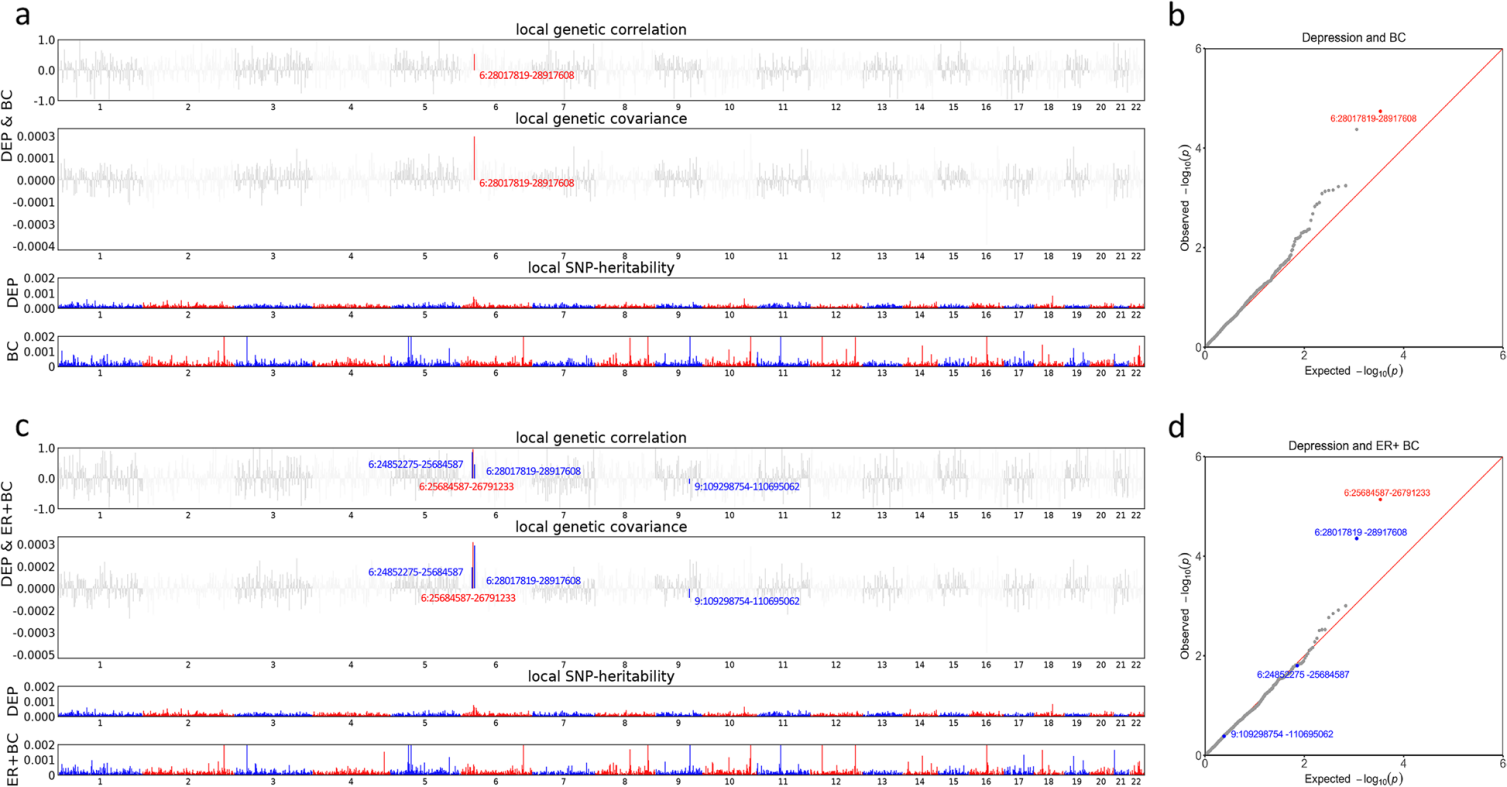
Local genetic correlation between depression and breast cancer. Red bars in Manhattan plots and red dots in QQ plots represent loci showing significant local genetic correlation with ρ-HESS after multiple testing adjustments (*P* < 0.05/1,703). Blue bars in Manhattan plots and blue dots in QQ plots represent additional loci showing a high probability of shared associations using GWAS-PW (PPA _3 > 0.5). **a** Manhattan plot showing the local genetic correlation, genetic covariance, and SNP-heritability estimated from ρ-HESS between depression and overall breast cancer. **b** QQ plot presenting region-specific *P*-values for ρ-HESS-estimated local genetic correlation between depression and overall breast cancer. **c** Manhattan plot showing the local genetic correlation, genetic covariance, and SNP-heritability estimated from ρ-HESS between depression and ER + breast cancer. **d** QQ plot presenting region-specific *P*-values for ρ-HESS-estimated local genetic correlation between depression and ER + breast cancer. DEP: depression; BC: breast cancer; ER: estrogen receptor

When the whole genome was partitioned, one genomic region at 6p22.1 (harboring a previous-reported depression locus *ZSCAN12*) was identified as contributing a significant local genetic correlation to depression and overall BC (*P*_ρ-HESS_ = 1.83 × 10^–5^). When examining cancer subtypes, a region at 6p22.2 (harboring a previous-reported depression locus *ABT1*) was identified as contributing to both depression and ER + BC (*P*_ρ-HESS_ = 7.30 × 10^–6^), which was further replicated by GWAS-PW (PPA_3 = 0.73). Additionally, GWAS-PW identified three regions showing high probability of shared associations between depression and ER + BC, including two regions near 6p22.2 (6p22.1, PPA_3 = 0.71; 6p22.3–22.2, PPA_3 = 0.89), and one region at 9q31.2 (PPA_3 = 0.52, harboring a previous-reported BC locus *KLF4*) (Fig. 2). No significant shared region was found for depression with ER − BC.

### Cross-trait meta-analysis and pleiotropic loci

Motivated by the significant genetic overlap observed for depression and BC, we continued to explore at the individual variant level. In total, 17 independent loci reached genome-wide significance in CPASSOC (*P*_CPASSOC_ < 5 × 10^–8^ and *P*_depression_ < 1 × 10^–3^ and *P*_BC_ < 1 × 10^–3^), including 12 loci shared between depression and overall BC, and nine loci shared between depression and ER + BC (Table 3). No significant shared locus was found for depression with ER– BC.

**Table 3:** Genome-wide significant loci shared between depression and breast cancer in cross-trait meta-analysis (*P*_CPASSOC_ < 5 × 10^–8^ and *P*_single-trait_ < 1 × 10^–3^)

**SNP**	**CHR:Position**	**A1**	**A2**	**BETA**		***P***_**single-trait**_		***P***_**CPASSOC**_	**Linear closest gene**^**a**^	**Interacting gene**^**b**^
				**BC**	**Depression**	**BC**	**Depression**			
*Depression and overall breast cancer*										
rs78955704	chr5:45072777-45968819	A	T	0.10	0.04	3.57×10^-10^	4.72×10^-04^	1.29×10^-09^	*HCN1*	..
rs68006638	chr6:25219582-26090025	A	G	-0.06	-0.04	4.98×10^-07^	2.13×10^-08^	1.44×10^-12^	..	..
rs35400317	chr6:26095643-27091661	T	C	-0.06	-0.05	5.35×10^-08^	3.87×10^-14^	1.41×10^-19^	*ABT1*	*BTN3A2* and other 11
rs35768595	chr6:27103654-27626631	T	C	-0.06	-0.05	1.45×10^-07^	8.21×10^-14^	8.42×10^-19^	..	*ZNF184* and other 30
rs17693963	chr6:27219491-28173219	A	C	0.05	0.06	2.21×10^-06^	2.31×10^-16^	8.96×10^-21^	*RP1-97D16.1*	*PRSS16* and other 44
rs67981811	chr6:27854963-28411941	C	G	0.06	0.06	4.17×10^-07^	1.14×10^-18^	6.52×10^-24^	*ZSCAN12*	..
rs9263717	chr6:31078804-31321919	A	G	-0.03	-0.02	3.64×10^-06^	2.32×10^-05^	1.92×10^-08^	*PSORS1C1*, *CR753819.4*, *BX927139.2*, *CR938714.1*	*DDR1* and other 19
rs56101042	chr14:103229696-103387971	A	G	0.03	-0.03	9.58×10^-04^	5.06×10^-07^	6.53×10^-09^	*TRAF3*, *snoU13*	*TRAF3* and other 4
rs4906340	chr14:103833385-104174123	A	G	-0.02	-0.03	3.37×10^-04^	1.06×10^-09^	4.22×10^-12^	*RP11-73ƒM18.2*, *APOPT1*, *KLC1*	*KLC1* and other 2
rs2403907	chr21:16488615-16578800	A	C	-0.08	0.02	1.38×10^-33^	6.19×10^-04^	1.55×10^-33^	..	..
rs5762586	chr22:28540492-29090363	A	T	-0.04	-0.01	3.88×10^-09^	5.40×10^-04^	1.43×10^-08^	*TTC28*	..
rs5757946	chr22:40809034-40809034	A	G	-0.04	-0.02	3.77×10^-12^	4.05×10^-04^	1.28×10^-11^	*SGSM3*, *MKL1*, *RP5-1042K10.13*	*MKL1* and other 1
*Depression and ER+ breast cancer*										
rs13198474	chr6:25419094-26090025	A	G	-0.08	-0.04	2.68×10^-07^	2.93×10^-08^	2.30×10^-11^	*SLC17A3*	..
rs45527431	chr6:26111271-27091661	A	G	0.08	0.05	6.74×10^-08^	3.83×10^-14^	3.92×10^-18^	*ABT1*	*BTN3A2* and other 11
rs35768595	chr6:27103654-27626631	T	C	-0.08	-0.05	7.16×10^-08^	8.21×10^-14^	1.38×10^-17^	..	..
rs17693963	chr6:27219491-28173219	A	C	0.08	0.06	2.95×10^-08^	2.31×10^-16^	8.85×10^-21^	*RP1-97D16.1*	*PRSS16* and other 44
rs67981811	chr6:27854963-28411941	C	G	0.08	0.06	4.40×10^-08^	1.14×10^-18^	3.14×10^-23^	*ZSCAN12*	..
rs7847927	chr9:110297639-110333432	C	G	0.07	-0.02	3.62×10^-20^	5.47×10^-04^	3.12×10^-19^	*RP11-438P9.2*	..
rs10843109	chr12:28288716-2857324	T	C	-0.08	0.03	1.60×10^-09^	5.10×10^-04^	8.32×10^-09^	*CCDC91*	..
rs1343607	chr13:53617781-54049489	A	G	-0.03	0.02	8.33×10^-04^	1.07×10^-07^	3.31×10^-09^	..	*OLFM4*
rs2403907	chr21:16488615-16582933	A	C	-0.10	0.02	2.95×10^-33^	6.19×10^-04^	1.91×10^-32^	..	..

Position is under build 37 (hg19)

^a^Linear closest genes of index SNPs were mapped by using VEP

^b^3D interacting genes of index SNPs were mapped by using 3DSNP

*SNP* Single nucleotide polymorphism, *CHR* Chromosome, *BC* Breast cancer, *ER* Estrogen receptor

Of these 17 pleiotropic SNPs, interestingly, SNP rs2403907 represented the strongest shared signal across both overall (*P*_CPASSOC_ = 1.55 × 10^–33^) and ER + BC (*P*_CPASSOC_ = 1.91 × 10^–32^), located at an intergenic region at 21q21.1. SNPs rs67981811 (overall BC: *P*_CPASSOC_ = 6.25 × 10^–24^; ER + BC:* P*_CPASSOC_ = 3.14 × 10^–23^) and rs17693963 (overall BC:* P*_CPASSOC_ = 8.96 × 10^–21^; ER + BC:* P*_CPASSOC_ = 8.85 × 10^–21^) were the second and the third strongest shared signals, both located at the 6p22.1 region that harbors a depression-associated risk locus [9].

In addition to “known” pleiotropic SNPs (SNPs significantly associated with both traits in previous GWASs, *N* = 6) or “single-trait-driven” pleiotropic SNPs (SNPs significantly associated with one of the two traits in previous GWASs, *N* = 10), we identified one novel shared SNP (re56101042) for depression and overall BC (*P*_CPASSOC_ = 6.53 × 10^–9^). This SNP is located at 14q32.32 near *snoU13* and *TRAF3*. The former gene represents a small nucleolar RNA recently implicated as a biomarker in vulnerability to brain injury [40], while the latter gene functions in immune modulation and inflammation via its regulation of nuclear factor-κB signaling [41], which has been implicated in the pathogenesis of both psychiatric disorders and tumorigenesis [1]. Four genes (*AMN*, *ANKRD9*, *MIR4309*, and *RCOR1*) were additionally identified to interact with rs56101042 through three-dimensional chromatin looping [25]. Detailed annotations for each SNP discovered by CPASSOC are shown in Additional file 3: Tables S4-S5.

### Identification of causal variants and colocalization

For each of the 17 pleiotropic SNPs, we further determined a 99% credible set of causal SNPs, providing targets for downstream experimental analysis (Additional file 3: Tables S6-S7). We found 413 candidate causal SNPs across all loci shared by depression and overall BC, and 282 candidate causal SNPs across all loci shared by depression and ER + BC. In particular, SNPs rs5757946 (22q13.1) and rs11049366 (12p11.22) were identified as having a posterior probability of 1.00 in the 99% credible set.

We also performed colocalization analysis to determine whether pleiotropic SNPs driving the associations in two traits were the same (Additional file 3: Table S8). A substantial proportion of shared loci (five out of the 12 loci shared by depression and overall BC; and five out of the nine loci shared by depression and ER + BC) colocalized at the same candidate SNPs (eight of which showed a PPH4 > 0.90), reinforcing shared causal associations.

### Transcriptome-wide association studies and shared genes

Results from tissue-specific TWAS revealed gene-level genetic overlap (single-trait results shown in Additional file 3: Table S9 and Additional file 1: Figure S2). After multiple corrections, a total of five genes were shared by depression and overall BC of which two were also shared by depression and ER + BC, enriched in six tissues including brain, blood, uterus, colon, artery and heart (Table 4). Four genes were located at pleiotropic loci identified in cross-trait meta-analysis, including *FLOT1* and *HLA-S* at 6p21.33, ENSG00000247934.4 at 12p11.22, and *GRAP2* at 22q13.1.

**Table 4:** Significant shared transcriptome-wide association analysis results between depression and breast cancer

Tissue	Ensembl gene ID	Gene symbol	CHR	BC			Depression		
				**Best.GWAS.ID**	**TWAS.Z**	***P***_**Bonferroni**_	**Best.GWAS.ID**	**TWAS.Z**	***P***_**Bonferroni**_
*Depression and overall breast cancer*									
Whole Blood	ENSG00000137312.14	*FLOT1*	6	rs3130975	-4.66	2.56 × 10^–02^	rs3130557	-6.15	6.10 × 10^–06^
Uterus	ENSG00000225851.1	*HLA-S*	6	rs3130975	4.30	4.07 × 10^–02^	rs3130557	4.84	3.07 × 10^–03^
Artery Coronary	ENSG00000247934.4	.	12	rs7297051	7.41	4.87 × 10^–10^	rs11049301	-4.44	3.48 × 10^–02^
Brain Cerebellum	ENSG00000168612.4	*ZSWIM1*	20	rs3746506	-4.60	3.15 × 10^–02^	rs9074	4.83	9.79 × 10^–03^
Heart Left Ventricle	ENSG00000100351.16	*GRAP2*	22	rs17001868	-4.87	6.69 × 10^–03^	rs139915	-4.50	3.93 × 10^–02^
*Depression and ER* + *breast cancer*									
Colon Transverse	ENSG00000137312.14	*FLOT1*	6	rs3130975	-4.49	4.68 × 10^–02^	rs3130557	-7.49	4.31 × 10^–10^
Artery Coronary	ENSG00000247934.4	.	12	rs7297051	5.50	1.49 × 10^–04^	rs11049301	-4.44	3.48 × 10^–02^

All these loci were TWAS significant (*P*_Bonferroni_ < 0.05) for both depression and breast cancer in at least one GTEx tissues

*TWAS* Transcriptome-wide association studies, *CHR* Chromosome, *Best.GWAS.ID* rsID of the most significant GWAS SNP in locus, *TWAS.Z* TWAS Z-score, *BC* Breast cancer, *ER* Estrogen receptor

While data on ENSG00000247934.4 was limited, it represents an antisense to *CCDC91* previously implicated in BC and brain morphology estimates (compared with data from GWAS Catalog [13]). Among other TWAS-significant shared genes, *FLOT1* was previously implicated in depression and aerodigestive squamous cell carcinoma. We also identified three novel genes, *HLA-S*, *ZSWIM1*, and *GRAP2*. As a pseudogene located at the major histocompatibility complex (MHC) region, *HLA-S* was reported to be associated with a broad range of conditions including schizophrenia, gastric, and hepatocellular cancers. By regulating T helper cells development, *ZSWIM1* plays a critical role in immune system [42], and signals a poor prognosis of ovarian cancer [43]. Similarly, *GRAP2* was implicated in T cell-mediated immune responses [44], as well as in medullary thyroid cancer and lung adenocarcinoma prognosis [45, 46].

### Mendelian randomization

**Fig. 3:**
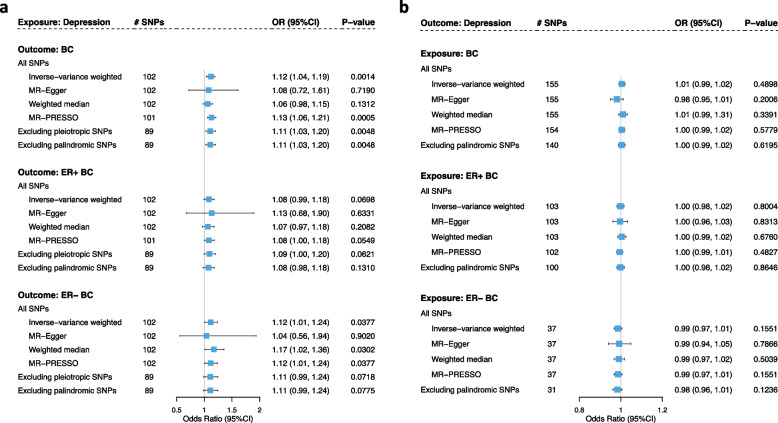
Bi-directional causal relationship underlying depression and breast cancer. **a** Estimates of causal effects for genetically predicted depression with overall breast cancer, ER + breast cancer, and ER– breast cancer, respectively. **b** Estimates of causal effects for genetically predicted overall breast cancer, genetically predicted ER + breast cancer, and genetically predicted ER– breast cancer with depression. Boxes represent the point estimates of causal effects, and error bars represent 95% confidence intervals. Inverse-variance weighted approach was adopted as the primary analysis. MR-Egger regression, weighted median, and MR-PRESSO approaches were adopted as sensitivity analysis. BC: breast cancer; ER: estrogen receptor

Finally, we conducted a two-sample MR analysis combining 102 GWAS-identified depression-associated SNPs (*F*-statistic ranging from 91.5 to 329.6 (Additional file 3: Table S1)) as an IV to examine a potential causal effect of depression on BC. Using IVW, genetic liability to depression was significantly associated with an increased risk of overall BC (odds ratio (OR) = 1.12, 95%CIs = 1.04–1.19, *P* = 1.40 × 10^–3^). The estimates remained directionally consistent in MR-Egger regression and the weighted median approach, despite larger statistical uncertainties (Fig. 3). No indication of horizontal pleiotropy was observed (*P*_MR-Egger intercept_ = 0.86). Sensitivity analyses excluding palindromic SNPs or pleiotropic SNPs supported the robustness of these results. Leave-one-out analysis demonstrated the absence of outlying variants (Additional file 1: Figure S3) which was validated by MR-PRESSO (OR = 1.13, 95%CIs = 1.06–1.21, *P* = 5.00 × 10^–4^). MVMR accounting for potential confounders generated similar results with even more pronounced magnitude and significance, suggesting a depression-BC causal relationship independent of common confounders (Additional file 1: Figure S4).

In subgroup analysis defined by ER status, we found a non-significant association of depression with ER + BC (IVW OR = 1.08, 95%CIs = 0.99–1.18, *P* = 6.98 × 10^–2^; *P*_MR-Egger intercept_ = 0.86), and a suggestive significant association of depression with ER– BC (IVW OR = 1.12, 95%CIs = 1.01–1.24, *P* = 3.77 × 10^–2^; *P*_MR-Egger intercept_ = 0.82), though neither withstood multiple testing corrections (Fig. 3). In MVMR, while a putative causal effect of depression on ER + BC was observed, the causal effect of depression with ER– BC attenuated to null after adjustment for each confounder, and none survived multiple corrections (Additional file 1: Figure S4).

No evidence of reverse causality was found such that genetically predicted BC did not seem to influence depression risk (IVW OR = 1.01, *P* = 0.49; MR-Egger OR = 0.98, *P* = 0.20; weighted median OR = 1.01, *P* = 0.34). Similar null effects were identified for ER + and ER– subtypes (Fig. 3).

## Discussion

To our knowledge, this is the largest observational and genetic analysis to systematically investigate the phenotypic association as well as the shared genetic architecture underlying depression and BC. Our observational study demonstrated a positive association of depression with BC risk, which was corroborated by findings from genetic analyses. We found evidence supporting a significant shared genetic basis, both globally and regionally, indicating shared biology between the two traits. This genetic overlap was further decomposed into both pleiotropy and causality, reflected by the pleiotropic loci identified in CPASSOC, the shared genes revealed by TWAS, and the putative causal relationship demonstrated by MR.

Overall, the genetic correlation between depression and BC was limited (${r}_{g}$ = 0.08) but statistically significant. In addition, significant local genetic correlations were found for several specific genomic regions including a locus at 9q31.2, and 4 loci at, or close to 6p22.1. Chr6q22.1 represents an important locus at MHC, a region well-known for containing multiple genes essential for the adaptive immune system as well as the anti-tumoral immune response [47]. The MHC region has been associated with depression in some prior studies [9–11, 48], with the strongest association located in the classical or extended class I region; moreover, a study has also identified a 66% down-regulation of MHC class I expression in BC tissues [49], collectively highlighting its role in the etiological connection underlying these two traits. Leveraging genotype imputation, a recent (also the largest) study on depression and classical MHC variants indicated an overall non-significant association [50], calling for additional efforts to dissect signals within the extended MHC.

Significant genetic overlap can be the result of biological or horizontal pleiotropy (in which a genetic variant affects multiple traits) and/or mediated or vertical pleiotropy (in which a genetic variant affects a trait via its effect on an intermediate trait) [51]. In our downstream analysis performed to explore these alternatives, 17 pleiotropic loci between depression and BC were identified, harboring genes which were previously implicated in neural development and brain functions (i.e., *HCN1*, *ABT1*, *ZSCAN12*, *ZNF184*, *KLC1*, *MKL1*, *SGSM3*, *CCDC91*), or biological processes related to tumor growth (i.e., *ABT1*, *ZSCAN12*, *ZNF184*, *KLC1*, *TTC28*, *MKL1*, *CCDC91*, *OLFM4*, *DDR1*). Notably, several shared loci were located at genes that play essential roles in immune, inflammatory, and stress responses (i.e., *PSORS1C1*, *TRAF3*, *APOPT1*, *PRSS16*, *BTN3A2*). Multiple genes showed strong evidence of colocalization, especially among those located near the MHC (i.e., *ABT1*, *BTN3A2*, *ZNF184*, *PRSS16*, *ZSCAN12*, *SLC17A3*).

One advantage of meta-analyzing GWASs of different traits is that combining association evidence across multiple studies can reveal signals which might not have reached genome-wide significance in a single-trait analysis. Indeed, we found a novel locus (lead SNP rs56101042) shared between depression and BC, only reported previously as associated with allergic disease [52]. While its direct involvement in the development of depression or BC is not known, this SNP interacts with several genes that have already been reported (i.e., *TRAF3* and *RCOR1* [53, 54]), or that could play a role in the pathogenesis of the two traits (i.e., *AMN*, *ANKRD9*, and *MIR4309*). Encoding a transmembrane protein named amnionless, *AMN* is involved in the plasma membrane transport of Cubilin, and may influence cancer progression through the Cubilin-FGF8 (fibroblast growth factor 8) interaction [55]. *AMN* may also function in psychiatric illness given its role in the vitamin B_12_ transportation into nervous system [56]. *ANKRD9* encodes an ankyrin repeat domain protein which was implicated in drug-induced mental disorder [57], and recognized as a tumor suppressor in gastric cancer among Asians [58]. *MIR4309* has been found to be significantly elevated among patients with disordered sleep (a core symptom of depression) [59], as well as up-regulated in tissues of gastric cancer [60]. Further experimental studies would be needed to provide more detailed functional annotation of rs56101042, particularly in relation to the onset of depression and BC.

By integrating data from GWAS and GTEx tissue expression, TWAS suggested shared mechanistic hypotheses between depression and BC on a gene-tissue pair level. The four loci identified in both CPASSOC and TWAS analysis implicate common biological mechanisms in depression and BC regulation, involving cell proliferation [61], brain structure [62, 63], and immune response [44]. In addition to tissues of brain, uterus, and blood (well-recognized as relevant to depression and/or BC [64–66]), our TWAS reported shared regulatory features in the digestive (i.e., colon transverse) and cardiovascular systems (i.e., artery coronary and hear left ventricle), suggesting the possibility of shared pathways extending to a wider range of organs. A growing body of literature has shown that functional disorders of the digestive system may play a critical role in a variety of psychiatric disorders including depression. One of the speculated mechanisms is the communication of gut microbiome with the brain through the brain-gut axis [67]. Interestingly, gut microbiome may also affect breast carcinogenesis by promoting anti-tumor immunity and/or modulating systemic estrogen levels [68]. As another major cause of mortality and morbidity, cardiovascular disease shares a number of common risk factors with both conditions, and represents one of the most prevalent comorbidities for depression and BC in clinical settings [69, 70]. More studies are needed to clarify the role of these hypothesized mechanisms.

To date, epidemiological evidence regarding the depression-BC relationship has been inconclusive, but our observational and MR analyses showed consistent evidence for a putative causal role of depression in BC among European populations. Our MR results extended existing MR analyses [71, 72] with substantially improved accuracy and precision of causal estimates by applying: (i) the most updated GWAS of overall BC; (ii) a broader range of complementary analysis to validate the MR assumptions; and (iii) an extensive MVMR design to evaluate the independent effect of depression. The estimated causal effects remained directionally consistent across a variety of sensitivity analyses, as well as after adjustment of multiple confounders, supporting the robustness of our MR findings. When extended to BC subtypes, we found consistently positive associations with greater statistical uncertainties. Future studies with larger sample sizes are warranted to establish or rule out a potential causal link in a subtype-specific manner.

Several limitations need to be acknowledged. Using data of European ancestry populations restricted the generalizability of our results, and future studies including more diverse ancestries are needed. The broad depression phenotype used in the current study is regarded as a relatively non-specific index of vulnerability to psychological distress, rather than highly specific for MD [73]. However, we performed sensitivity analysis restricting to clinically ascertained MD cases and identified consistent results (fully adjusted observational analysis using only ICD-10 codes F32 and F33 for MD diagnosis: HR = 1.091, 95%CIs = 0.946–1.258; genetic correlation and MR analyses applying an older MD GWAS [10] involving only clinically defined MD cases: ${r}_{g}$ = 0.07, *P* = 3.00 × 10^–4^; OR_MR_ = 1.08, *P* = 4.30 × 10^–2^). Additionally, due to limited data availability at the time of conducting the analysis, we were unable to utilize sex-specific GWAS data of depression to match with the female-specific outcome BC. However, sex-heterogeneity did not seem to play a significant role when using female-specific depression GWAS summary statistics which now become available [74]. Although underpowered, female-specific genetic correlation analysis and MR analysis yielded statistically significant and directionally consistent results (${r}_{g}$ = 0.09, *P* = 1.60 × 10^−3^; OR_IVW_ = 1.11, *P* = 0.04). Using sufficiently powered female-specific data would be valuable for future studies. Although depression is highly heterogeneous, we did not investigate depression subtypes (i.e., typical vs. atypical) or additional BC subtypes due to limited well-powered GWAS available for these phenotypes. Finally, validation of the study findings in additional follow-up cohorts and GWAS is needed. Future in-depth experimental works are also warranted to better understand pathophysiological mechanisms that may underlie genetic overlap between depression and BC.

## Conclusions

In sum, the current study advances our understanding of previously reported associations between depression and BC by providing evidence of genetic correlation, pleiotropic loci, and a possible causal effect of depression on BC. Our findings further suggest potential biological mechanisms linking these disorders and may have implications for future studies aimed reducing BC risk.

## Supplementary Information

Additional file 1: Figure S1. Graphical abstract. Figure S2. Number of TWAS significant genes for depression, overall BC, and subtype-specific BC. Figure S3. Box plot of betas in leave-one-out analysis. Figure S4. Results of multi-variable Mendelian randomization analysis.

Additional file 2. STROBE and STROBE-MR checklists.

Additional file 3: Table S1. Depression IVs. Table S2. Summary of GWAS data. Table S3. Baseline characteristics of UK Biobank participants. Tables S4-S5. Annotation of CPASSOC-identified SNPs. Tables S6-S7. 99% credible sets of CPASSOC-identified SNPs. Table S8. Results from colocalization analysis for CPASSOC-identified SNPs. Table S9. TWAS significant gene-tissue pairs for depression, overall BC, and subtype-specific BC.

## Data Availability

Data from UK Biobank is available for open access to scientific researcher (www.biobank.ac.uk). Depression GWAS summary statistics (without 23andMe) is publicly available from https://datashare.ed.ac.uk/handle/10283/3203. GWAS summary statistics for overall and subtype-specific BCs are publicly available from https://bcac.ccge.medschl.cam.ac.uk/bcacdata/oncoarray/oncoarray-and-combined-summary-result/.

## Abbreviations

BC: Breast cancer
BMI: Body mass index
CIs: Confidence intervals
CPASSOC: Cross-Phenotype Association
ER: Estrogen receptor
GTEx: Genotype-Tissue Expression
GWAS: Genome-wide association study
GWAS-PW: Pairwise-GWAS
HESS: Heritability Estimator from Summary Statistics
HR: Hazard ratio
IV: Instrumental variable
IVW: Inverse-variance weighted
LD: Linkage disequilibrium
LDSC: Linkage disequilibrium Score Regression
MD: Major depression
MHC: Major histocompatibility complex
MR: Mendelian randomization
MR-PRESSO: MR-Pleiotropy Residual Sum and Outlier
MVMR: Multi-variable Mendelian randomization
OR: Odds ratio
PPA: Posterior probabilities of association
*r*_*g*_: Genetic correlation
RR: Relative risk
SNP: Single nucleotide polymorphism
TWAS: Transcriptome-wide association study
UKBB: UK Biobank
VEP: Ensembl Variant Effect Predictor
