# Supplementary material for: Investigating the relationship between depression and breast cancer: observational and genetic analyses
Source: BMC Med. 2023 May 4;21:170. doi: 10.1186/s12916-023-02876-w (PMC10161423; doi:10.1186/s12916-023-02876-w)
Supplement: Supplementary file 1 — Additional file 1: Figure S1. Graphical abstract. Figure S2. Number of TWAS significant genes for depression, overall BC, and subtype-specific BC. Figure S3. Box plot of betas in leave-one-out analysis. Figure S4. Results of multi-variable Mendelian randomization analysis. [file 12916_2023_2876_MOESM1_ESM.pdf]

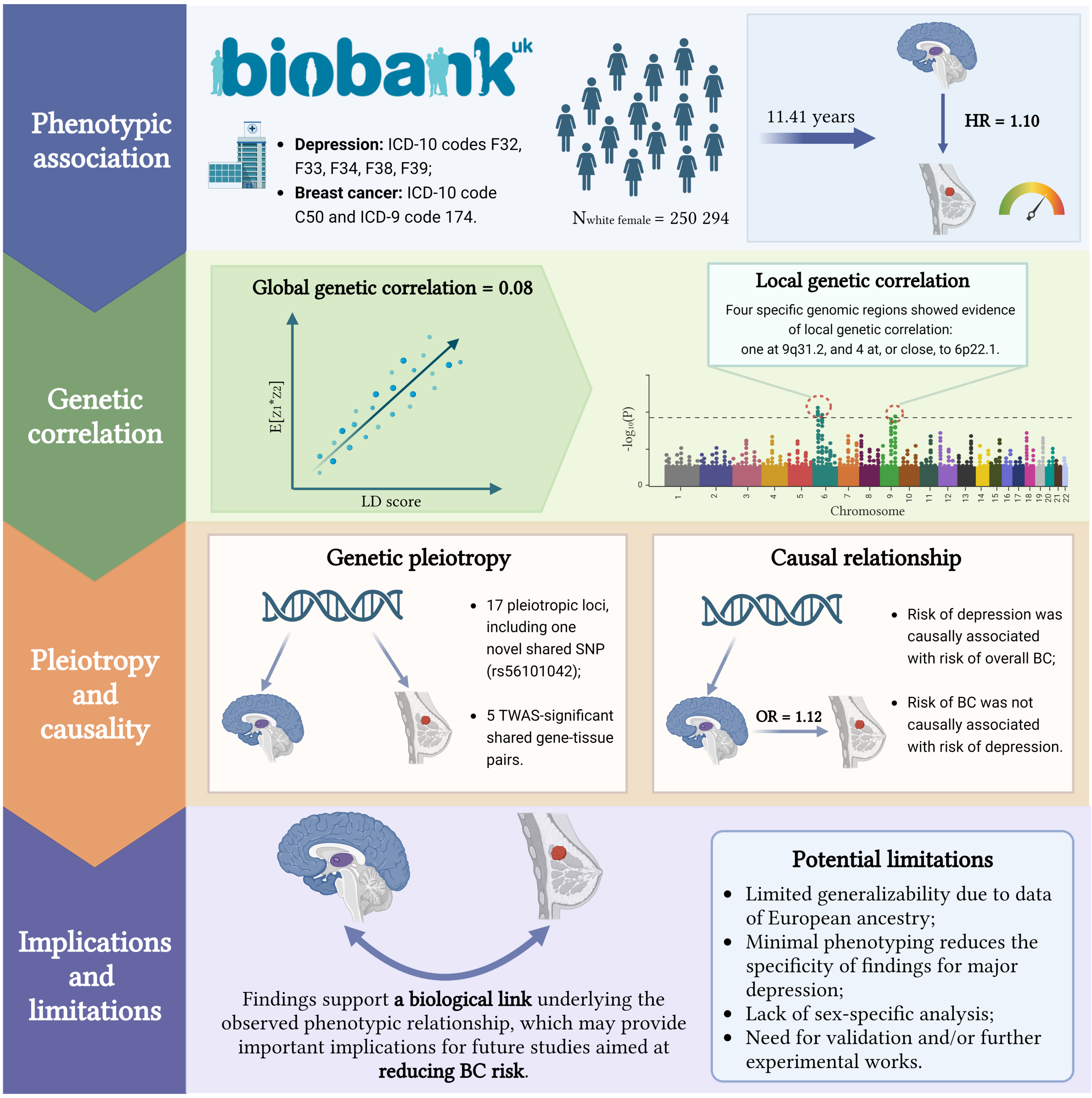

Figure S1. Graphical representation of findings from the present study.

ICD: international classification of diseases; LD: linkage disequilibrium; TWAS: transcriptome-wide association study; BC: breast cancer.

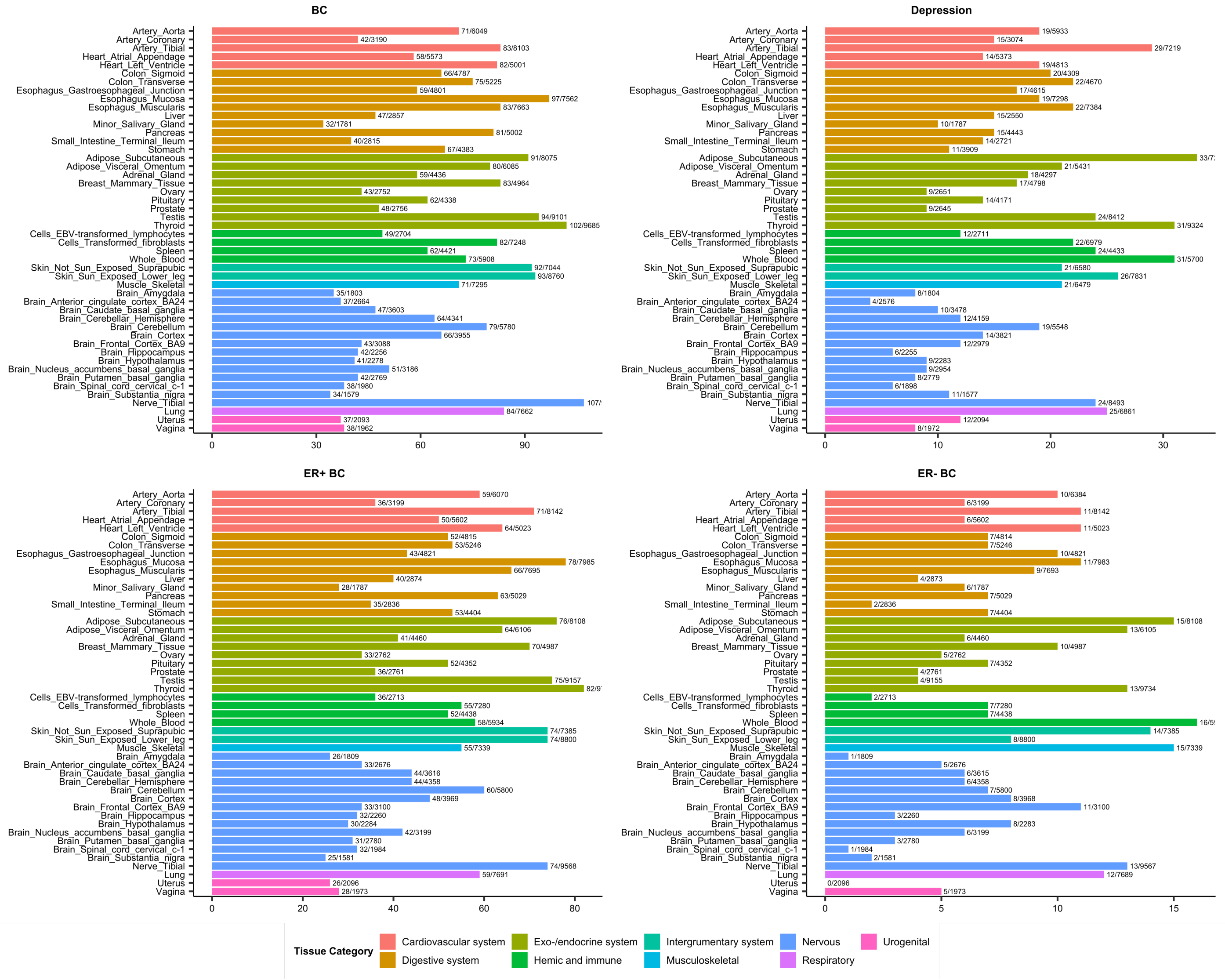

Figure S2. Number of TWAS significant genes for depression and breast cancer across 49 GTEx tissues (version 8). The X axis shows the count of genes from tissues in the GTEx database meeting significance thresholds for multiple testing for each trait. The Y axis lists GTEx tissues. Colors represent different tissue categories. The null hypothesis of TWAS is no expression-trait association (or genetic correlation between expression and a trait) conditional on the observed GWAS statistics at the corresponding locus. The total number of TWAS gene-tissue pairs being tested is ~290,000 across 49 GTEx tissues.

TWAS: transcriptome-wide association study; BC: breast cancer; ER: estrogen receptor.

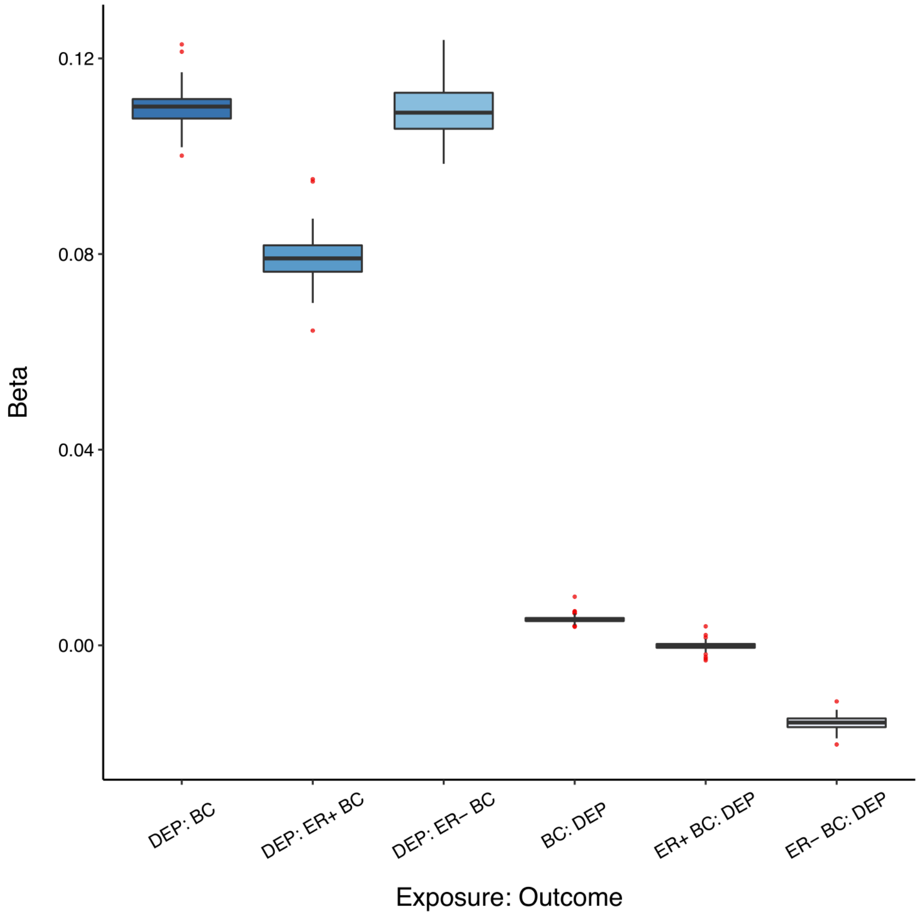

Figure S3. Box plot of betas in leave-one-out analysis where one SNP was removed at a time and inverse-variance weighted analysis was conducted based on the remaining SNPs.

Beta: effect allele beta coefficient; BC: breast cancer; DEP: depression; ER: estrogen receptor.

Exposure: Depression	# SNPs	OR (95%CI)	P–value
**Outcome: BC**			
adjNone	102	1.12 (1.04–1.19)	0.0014
adjBody_Mass_Index	95	1.13 (1.06–1.22)	0.0007
adjSmoking_Initiation	95	1.14 (1.05–1.24)	0.0006
adjDrinks_per_Week	95	1.12 (1.04–1.20)	0.0020
adjPhysical_Activity	95	1.12 (1.04–1.20)	0.0020
adjSleep_Duration	95	1.12 (1.05–1.20)	0.0013
adjEducational_Attainment	95	1.10 (1.03–1.18)	0.0093
adjAll	95	1.15 (1.06–1.26)	0.0010
**Outcome: ER+ BC**			
adjNone	102	1.08 (0.99–1.18)	0.0698
adjBody_Mass_Index	95	1.12 (1.02–1.22)	0.0147
adjSmoking_Initiation	95	1.11 (0.99–1.23)	0.0631
adjDrinks_per_Week	95	1.08 (0.99–1.18)	0.0705
adjPhysical_Activity	95	1.09 (1.00–1.19)	0.0559
adjSleep_Duration	95	1.10 (1.01–1.20)	0.0335
adjEducational_Attainment	95	1.08 (0.99–1.19)	0.0794
adjAll	95	1.13 (1.01–1.26)	0.0291
**Outcome: ER– BC**			
adjNone	102	1.12 (1.01–1.24)	0.0377
adjBody_Mass_Index	95	1.11 (0.99–1.24)	0.0688
adjSmoking_Initiation	95	1.10 (0.97–1.26)	0.1351
adjDrinks_per_Week	95	1.11 (0.99–1.23)	0.0679
adjPhysical_Activity	95	1.09 (0.98–1.22)	0.1053
adjSleep_Duration	95	1.10 (0.99–1.22)	0.0895
adjEducational_Attainment	95	1.09 (0.97–1.21)	0.1362
adjAll	95	1.09 (0.96–1.25)	0.1952

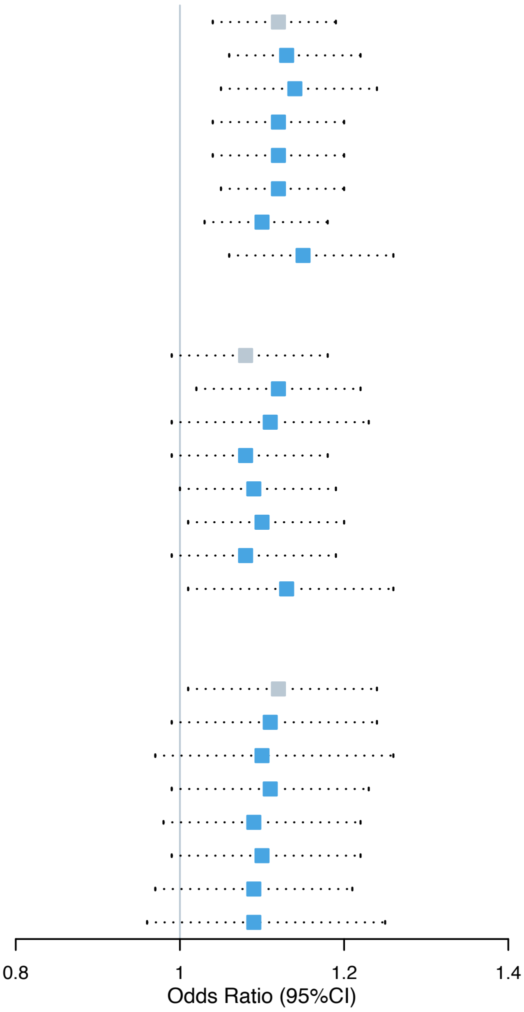

Figure S4. Independent effects of genetically predicted depression on overall breast cancer, ER+ breast cancer, and ER– breast cancer after adjusting for each potential confounder separately and together using multi-variable Mendelian randomization. The y-axis details the genetically predicted confounder for which adjustment was made. Boxes represent the point estimates of the causal effects of genetically predicted depression on breast cancer, and error bars represent 95% confidence intervals.

adjNone: the point estimates of the causal effects of genetically predicted depression on breast cancer using univariable Mendelian randomization; BC: breast cancer; ER: estrogen receptor.
