# Supplementary material for: Investigating the relationship between depression and breast cancer: observational and genetic analyses
Source: BMC Med. 2023 May 4;21:170. doi: 10.1186/s12916-023-02876-w (PMC10161423; doi:10.1186/s12916-023-02876-w)
Supplement: Supplementary file 2 — Additional file 2. STROBE and STROBE-MR checklists. [file 12916_2023_2876_MOESM2_ESM.pdf]

STROBE Statement—checklist of items that should be included in reports of observational studies

	Item No.	Recommendation	Page No.	Relevant text from manuscript
**Title and abstract**	1	(*a*) Indicate the study's design with a commonly used term in the title or the abstract	3	"We first evaluated phenotypic association using longitudinal follow-up data from the UK Biobank …"
		(*b*) Provide in the abstract an informative and balanced summary of what was done and what was found	3	"Observational analysis suggested an increased hazard of BC in depression patients …"
**Introduction**				
Background/rational e	2	Explain the scientific background and rationale for the investigation being reported	4	"…One example of this phenotypic association is depression and breast cancer … Hypothesized underlying biological mechanisms include … Population-based evidence regarding the depression-BC relationship, however, remains inconsistent. …"
Objectives	3	State specific objectives, including any prespecified hypotheses	5	"…Here we apply these methods to dissect the genetic and phenotypic relationships between depression and BC. …"
**Methods**				
Study design	4	Present key elements of study design early in the paper	5	"We first used data from UK Biobank (UKBB) to evaluate the phenotypic association. …"
Setting	5	Describe the setting, locations, and relevant dates, including periods of recruitment, exposure, follow-up, and data collection	5	"UKBB is a large-scale prospective cohort study with … when recruited in 2006–2010"
Participants	6	(*a*) *Cohort study*—Give the eligibility criteria, and the sources and methods of selection of participants. Describe methods of follow-up<br>*Case-control study*—Give the eligibility criteria, and the sources and methods of case ascertainment and control selection. Give the rationale for the choice of cases and controls<br>*Cross-sectional study*—Give the eligibility criteria, and the sources and methods of selection of participants	5	"UKBB is a large-scale prospective cohort study with … 503,317 participants consented to join the study cohort and visited an assessment center, among which we only considered women of European descent … After excluding 6,856 participants with a history of BC at baseline … 250,294 participants were finally included …" (Figure 1)
		(*b*) *Cohort study*—For matched studies, give matching criteria and number of exposed and unexposed<br>*Case-control study*—For matched studies, give matching criteria and the number of controls per case	NA	NA
Variables	7	Clearly define all outcomes, exposures, predictors, potential confounders, and effect modifiers. Give diagnostic criteria, if applicable	5, 7	"…We defined a diagnosis of depression as … and a diagnosis of BC as … After excluding … 250,294

				participants were finally included … We used three sets of adjustments to minimize the role of confounding …”
Data sources/ measurement	8*	For each variable of interest, give sources of data and details of methods of assessment (measurement). Describe comparability of assessment methods if there is more than one group	5	“…Data-Field: 41202 and Data-Field: 41204 …”
Bias	9	Describe any efforts to address potential sources of bias	7	“… We used three sets of adjustments to minimize the role of confounding …”
Study size	10	Explain how the study size was arrived at	(Figure 1)	
Quantitative variables	11	Explain how quantitative variables were handled in the analyses. If applicable, describe which groupings were chosen and why	(Table S3, footnote)	
Statistical methods	12	(*a*) Describe all statistical methods, including those used to control for confounding	7	“We constructed a Cox proportional hazards regression model with … We used three sets of adjustments to minimize the role of confounding …”
		(*b*) Describe any methods used to examine subgroups and interactions	NA	NA
		(*c*) Explain how missing data were addressed	NA	NA
		(*d*) *Cohort study*—If applicable, explain how loss to follow-up was addressed<br>*Case-control study*—If applicable, explain how matching of cases and controls was addressed<br>*Cross-sectional study*—If applicable, describe analytical methods taking account of sampling strategy	NA	NA
		(*e*) Describe any sensitivity analyses	17	“…we performed sensitivity analysis restricting to clinically ascertained MD cases and identified consistent results …”
**Results**				
Participants	13*	(a) Report numbers of individuals at each stage of study—eg numbers potentially eligible, examined for eligibility, confirmed eligible, included in the study, completing follow-up, and analysed	(Figure 1)	
		(b) Give reasons for non-participation at each stage	5	“… among which we only considered women of European descent … After excluding 6,856 participants with a history of BC at baseline…”
		(c) Consider use of a flow diagram	(Figure 1)	
Descriptive data	14*	(a) Give characteristics of study participants (eg demographic, clinical, social) and information on exposures and potential confounders	(Figure S3)	
		(b) Indicate number of participants with missing data for each variable of interest	NA	NA
		(c) *Cohort study*—Summarise follow-up time (eg, average and total amount)	11	“…In total, participants were followed for 3,014,168 person-years (11.41 ± 2.95 years).”
Outcome data	15*	Cohort study—Report numbers of outcome events or summary measures over time	11	“…during which 529 depression patients and 9,516 depression-free individuals developed BC…”
		Case-control study—Report numbers in each exposure category, or summary measures of exposure		

		Cross-sectional study—Report numbers of outcome events or summary measures		
Main results	16	(*a*) Give unadjusted estimates and, if applicable, confounder-adjusted estimates and their precision (eg, 95% confidence interval). Make clear which confounders were adjusted for and why they were included	11	"…we observed a positive association between depression and risk of BC (hazard ratio (HR) = 1.075, 95%CIs = 0.963-1.199) … The effect was strengthened when additionally adjusted for …"
		(*b*) Report category boundaries when continuous variables were categorized	NA	NA
		(*c*) If relevant, consider translating estimates of relative risk into absolute risk for a meaningful time period	NA	NA
Other analyses	17	Report other analyses done—eg analyses of subgroups and interactions, and sensitivity analyses	17	"…we performed sensitivity analysis restricting to clinically ascertained MD cases and identified consistent results …"
**Discussion**				
Key results	18	Summarise key results with reference to study objectives	14	"… Our observational study demonstrated a positive association of depression with BC risk …"
Limitations	19	Discuss limitations of the study, taking into account sources of potential bias or imprecision. Discuss both direction and magnitude of any potential bias	17-18	"… Several limitations need to be acknowledged …"
Interpretation	20	Give a cautious overall interpretation of results considering objectives, limitations, multiplicity of analyses, results from similar studies, and other relevant evidence	17	"… our observational and MR analyses showed consistent evidence for a putative causal role of depression in BC among European populations …"
Generalisability	21	Discuss the generalisability (external validity) of the study results	17	"…Using data of European ancestry populations restricted the generalizability of our results, … The broad depression phenotype used in the current study is regarded as a relatively non-specific index of vulnerability to psychological distress, rather than highly specific for MD … we did not investigate depression subtypes … or additional BC subtypes …"
**Other information**				
Funding	22	Give the source of funding and the role of the funders for the present study and, if applicable, for the original study on which the present article is based	20	"This study was supported by funds from…"

*Give information separately for cases and controls in case-control studies and, if applicable, for exposed and unexposed groups in cohort and cross-sectional studies.

Note: An Explanation and Elaboration article discusses each checklist item and gives methodological background and published exampl es of transparent reporting. The STROBE checklist is best used in conjunction with this article (freely available on the Web sites of PLoS Medicine at http://www.plosmedicine.org/, Annals of Internal Medicine at http://www.annals.org/, and Epidemiology at http://www.epidem.com/). Information on the STROBE Initiative is available at www.strobe-statement.org.

STROBE-MR checklist of recommended items to address in reports of Mendelian randomization studies[1 2]

Item No.	Section		Checklist item	Page No.	Relevant text from manuscript
1	**TITLE and ABSTRACT**		Indicate Mendelian randomization (MR) as the study's design in the title and/or the abstract if that is a main purpose of the study	3	"… We aimed to comprehensively characterize the phenotypic and genetic relationships between depression and BC. … Bi-directional Mendelian randomization suggested …"
	**INTRODUCTION**				
2	**Background**		Explain the scientific background and rationale for the reported study. What is the exposure? Is a potential causal relationship between exposure and outcome plausible? Justify why MR is a helpful method to address the study question	4	"… Population-based evidence regarding the depression-BC relationship, however, remains inconsistent. … One way to disentangle these conflicting findings is to investigate the potential genetic underpinnings of comorbid disorders. … These results suggest that depression and BC may be linked by shared biology, though the extent and nature of such links remains unclear. …"
3	**Objectives**		State specific objectives clearly, including pre-specified causal hypotheses (if any). State that MR is a method that, under specific assumptions, intends to estimate causal effects	5	"… Here we apply these methods to dissect the genetic and phenotypic relationships between depression and BC. …, we quantified phenotypic association, global and local genetic correlations, pleiotropic loci, and potential causal relationships. …"
	**METHODS**				
4	**Study design and data sources**		Present key elements of the study design early in the article. Consider including a table listing sources of data for all phases of the study. For each data source contributing to the analysis, describe the following:	(Figure 1 and Table S2)	
		a)	Setting: Describe the study design and the underlying population, if possible. Describe the setting, locations, and relevant dates, including periods of recruitment, exposure, follow-up, and data collection, when available.	5-6	"GWAS summary data for depression was obtained from … The largest available GWAS summary data for overall BC was obtained from … Details on the characteristics of each included data set are presented in Additional file 1: Table S2. …"
		b)	Participants: Give the eligibility criteria, and the sources and methods of selection of participants. Report the sample size, and whether any power or sample size calculations were carried out prior to the main analysis	5-6	"GWAS summary data for depression was obtained from … The largest available GWAS summary data for overall BC was obtained from … Details on the

					characteristics of each included data set are presented in Additional file 1: Table S2. …”
		c)	Describe measurement, quality control and selection of genetic variants	5-6	“Independent trait-associated genome-wide significant single nucleotide polymorphisms (SNPs) were identified at … we determined IVs as the lead SNPs reaching genome-wide significance ($P < 5\times10^{-8}$) after …”
		d)	For each exposure, outcome, and other relevant variables, describe methods of assessment and diagnostic criteria for diseases	6	“…The phenotypes ranged from self-reported help-seeking for problems with “nerves, anxiety, tension or depression” (termed “broad depression”) (51.8%), self-reported clinical diagnosis of major depression (MD) (30.7%), and clinically ascertained diagnosis of MD (17.5%). … 133,384 clinically ascertained BC cases and 113,789 controls, …”
		e)	Provide details of ethics committee approval and participant informed consent, if relevant	NA	NA
5	**Assumptions**		Explicitly state the three core IV assumptions for the main analysis (relevance, independence and exclusion restriction) as well assumptions for any additional or sensitivity analysis	10	“Additional sensitivity analyses were conducted to validate MR model assumptions (i.e., relevance, independence, and exclusion restriction), including: …”
6	**Statistical methods: main analysis**		Describe statistical methods and statistics used		
		a)	Describe how quantitative variables were handled in the analyses (i.e., scale, units, model)	NA	NA
		b)	Describe how genetic variants were handled in the analyses and, if applicable, how their weights were selected	6	“Independent trait-associated genome-wide significant single nucleotide polymorphisms (SNPs) were identified at … we determined IVs as the lead SNPs reaching genome-wide significance ($P < 5\times10^{-8}$) after …”
		c)	Describe the MR estimator (e.g. two-stage least squares, Wald ratio) and related statistics. Detail the included covariates and, in case of two-sample MR, whether the same covariate set was used for adjustment in the two samples	9-10	“We used the random-effect inverse-variance weighted (IVW) approach as the primary approach. … Additional sensitivity analyses were conducted …”
		d)	Explain how missing data were addressed	NA	NA
		e)	If applicable, indicate how multiple testing was addressed	10	“… we defined a significant causal estimate as significant in IVW (Bonferroni corrected P-value < 0.05/3, number of overall BC and subtypes) ….”

7	**Assessment of assumptions**	Describe any methods or prior knowledge used to assess the assumptions or justify their validity	10	"… To reduce biased estimate due to pleiotropic effects of genetic instruments, we adopted two complementary methods: …"
8	**Sensitivity analyses and additional analyses**	Describe any sensitivity analyses or additional analyses performed (e.g. comparison of effect estimates from different approaches, independent replication, bias analytic techniques, validation of instruments, simulations)	10	"Additional sensitivity analyses were conducted to validate MR model assumptions (i.e., relevance, independence, and exclusion restriction), including: …"
9	**Software and pre-registration**			
	a)	Name statistical software and package(s), including version and settings used	10	"MR analyses were conducted using packages "TwoSampleMR" (version 0.5.4), "MRPRESSO" (version 1.0), and "MendelianRandomization" (version 0.7.0) in software R (version 4.1.0)."
	b)	State whether the study protocol and details were pre-registered (as well as when and where)	NA	NA
	**RESULTS**			
10	**Descriptive data**			
	a)	Report the numbers of individuals at each stage of included studies and reasons for exclusion. Consider use of a flow diagram	NA	NA
	b)	Report summary statistics for phenotypic exposure(s), outcome(s), and other relevant variables (e.g. means, SDs, proportions)	NA	NA
	c)	If the data sources include meta-analyses of previous studies, provide the assessments of heterogeneity across these studies	NA	NA
	d)	For two-sample MR:<br>i. Provide justification of the similarity of the genetic variant-exposure associations between the exposure and outcome samples<br>ii. Provide information on the number of individuals who overlap between the exposure and outcome studies	5-6<br>6	"…GWAS summary data for depression was obtained from … all of European ancestry … GWAS summary data for overall BC was obtained from … all of European ancestry …"<br>"… No overlapping participating studies were shared between the BCs and depression GWASs."
11	**Main results**			
	a)	Report the associations between genetic variant and exposure, and between genetic variant and outcome, preferably on an interpretable scale	(Table S1)	

		b)	Report MR estimates of the relationship between exposure and outcome, and the measures of uncertainty from the MR analysis, on an interpretable scale, such as odds ratio or relative risk per SD difference	13-14	"… Using IVW, genetic liability to depression was significantly associated with an increased risk of overall BC (odds ratio (OR) = 1.12, 95%CIs = 1.04-1.19, P = 1.40×10^{-3}). …"
		c)	If relevant, consider translating estimates of relative risk into absolute risk for a meaningful time period	NA	NA
		d)	Consider plots to visualize results (e.g. forest plot, scatterplot of associations between genetic variants and outcome versus between genetic variants and exposure)	(Figure 3 and Figure S3)	
12	**Assessment of assumptions**				
		a)	Report the assessment of the validity of the assumptions	13-14	"… The estimates remained directionally consistent in …"
		b)	Report any additional statistics (e.g., assessments of heterogeneity across genetic variants, such as I^2, Q statistic or E-value)	13-14	"… No indication of horizontal pleiotropy was observed ($P_{\text{MR-Egger intercept}}$ = 0.86) …"
13	**Sensitivity analyses and additional analyses**				
		a)	Report any sensitivity analyses to assess the robustness of the main results to violations of the assumptions	13-14	"… The estimates remained directionally consistent in …"
		b)	Report results from other sensitivity analyses or additional analyses	17	"…we performed sensitivity analysis restricting to clinically ascertained MD cases and identified consistent results … female-specific … MR analysis yielded statistically significant and directionally consistent results …"
		c)	Report any assessment of direction of causal relationship (e.g., bidirectional MR)	14	"… No evidence of reverse causality was found such that genetically predicted BC did not seem to influence depression risk …"
		d)	When relevant, report and compare with estimates from non-MR analyses	17	"… our observational and MR analyses showed consistent evidence for a putative causal role of depression in BC among European populations …"
		e)	Consider additional plots to visualize results (e.g., leave-one-out analyses)	(Figure S2)	

	**DISCUSSION**			
14	**Key results**	Summarize key results with reference to study objectives	14-15	"… We found evidence supporting a significant shared genetic basis, … reflected by … and the putative causal relationship demonstrated by MR."
15	**Limitations**	Discuss limitations of the study, taking into account the validity of the IV assumptions, other sources of potential bias, and imprecision. Discuss both direction and magnitude of any potential bias and any efforts to address them	17-18	"… Several limitations need to be acknowledged … validation of the study findings in additional follow-up cohorts and GWAS is needed …"
16	**Interpretation**			
	a)	Meaning: Give a cautious overall interpretation of results in the context of their limitations and in comparison with other studies	17	"… Our MR results extended existing MR analyses with …"
	b)	Mechanism: Discuss underlying biological mechanisms that could drive a potential causal relationship between the investigated exposure and the outcome, and whether the gene-environment equivalence assumption is reasonable. Use causal language carefully, clarifying that IV estimates may provide causal effects only under certain assumptions	15-17	"…implicate common biological mechanisms in depression and BC regulation, involving cell proliferation, brain structure, and immune response. … "
	c)	Clinical relevance: Discuss whether the results have clinical or public policy relevance, and to what extent they inform effect sizes of possible interventions	18	"… and may have implications for future studies aimed reducing BC risk …"
17	**Generalizability**	Discuss the generalizability of the study results (a) to other populations, (b) across other exposure periods/timings, and (c) across other levels of exposure	17	"…Using data of European ancestry populations restricted the generalizability of our results, … The broad depression phenotype used in the current study is regarded as a relatively non-specific index of vulnerability to psychological distress, rather than highly specific for MD… we were unable to utilize sex-specific GWAS data of depression … we did not investigate depression subtypes … or additional BC subtypes …"
	**OTHER INFORMATION**			
18	**Funding**	Describe sources of funding and the role of funders in the present study and, if applicable, sources of funding for the databases and original study or studies on which the present study is based	20	"This study was supported by funds from…"
19	**Data and data sharing**	Provide the data used to perform all analyses or report where and how the data can be accessed, and reference these sources in the article. Provide the statistical code needed to	5-6	"GWAS summary data for depression was obtained from … The largest available GWAS summary data for overall BC was obtained from … Details on the

		reproduce the results in the article, or report whether the code is publicly accessible and if so, where		characteristics of each included data set are presented in Additional file 1: Table S2. …”
20	**Conflicts of Interest**	All authors should declare all potential conflicts of interest	20	“The authors declare that they have no competing interests.”

This checklist is copyrighted by the Equator Network under the Creative Commons Attribution 3.0 Unported (CC BY 3.0) license.

1. Skrivankova VW, Richmond RC, Woolf BAR, Yarmolinsky J, Davies NM, Swanson SA, et al. Strengthening the Reporting of Observational Studies in Epidemiology using Mendelian Randomization (STROBE-MR) Statement. JAMA. 2021;under review.
2. Skrivankova VW, Richmond RC, Woolf BAR, Davies NM, Swanson SA, VanderWeele TJ, et al. Strengthening the Reporting of Observational Studies in Epidemiology using Mendelian Randomisation (STROBE-MR): Explanation and Elaboration. BMJ. 2021;375:n2233.
